# Renoprotective and neuroprotective effects of enteric hydrogen generation from Si-based agent

**DOI:** 10.1038/s41598-020-62755-9

**Published:** 2020-04-03

**Authors:** Yuki Kobayashi, Ryoichi Imamura, Yoshihisa Koyama, Makoto Kondo, Hikaru Kobayashi, Norio Nonomura, Shoichi Shimada

**Affiliations:** 1 Institute of Scientific and Industrial Research, Osaka University, Osaka, Japan; 2 Department of Urology, Osaka University Graduate School of Medicine, Osaka, Japan; 3 Department of Neuroscience and Cell Biology, Osaka University Graduate School of Medicine, Osaka, Japan

**Keywords:** Drug development, Chronic kidney disease, Parkinson's disease, Nanoparticles

## Abstract

We have developed Si-based agent which can generate a large amount of hydrogen. Si-based agent continues generating hydrogen for more than 24 h by the reaction with water under conditions similar to those in bowels, i.e., pH8.3 and 36 °C, and generates ~400 mL hydrogen. To investigate beneficial effects for diseases associated with oxidative stress, Si-based agent is administered to remnant kidney rats and Parkinson’s disease mice. Rats are fed with control or Si-based agent-containing diet for 8 weeks. Si-based agent is found to greatly suppress the development of renal failure and the parameters of oxidative stress. Treatment with Si-based agent in a mouse model of hemi-Parkinson’s disease induced by 6-hydroxydopamine attenuated degeneration of dopaminergic neurons and prevented impairment of motor balance and coordination. These findings indicate that the Si-based agent shows renoprotective and neuroprotective effects presumably via suppression of oxidative stress by generation of hydrogen.

## Introduction

Hydroxyl radicals (OH radicals), i.e., one of reactive oxygen species (ROS), are continuously *in vivo* generated for various reasons, e.g., metabolism^1^, UV irradiation^2^, etc. OH radicals have the highest oxidation-reduction potential among ROS, i.e., singlet molecular oxygen (^1^O_2_), superoxide anion radical (O_2_^−^), and hydrogen peroxide (H_2_O_2_). Due to the high oxidizing power, OH radicals oxidize cells, DNA^3^, and lipid^4^, contributing to various diseases, e.g., cancer cell proliferation^5^, diabetes^6^, Alzheimer’s disease^7,8^, Parkinson’s disease (PD)^7,8^, renal failure^9^, etc. Oxidation of cells by OH radicals also accelerates aging^10,11^. Other ROS except OH radicals possess physiological functions, e.g., H_2_O_2_ and O_2_^−^ are used by immune cells. Therefore, it is important to eliminate only OH radicals without removal of other ROS. Hydrogen reacts only with OH radicals among ROS and therefore, hydrogen can prevent diseases involving oxidative stress without side-effects. One of the methods is intake of hydrogen-rich water^12,13^. However, even in the case of saturated hydrogen concentration of 1.6 ppm, only 18 mL hydrogen gas is included in 1 L hydrogen-rich water. Even if hydrogen in hydrogen-rich water is absorbed in the body and transferred to organs, the hydrogen concentration there returns to the initial value in a short time, e.g., 1 h^14^. Therefore, the effect of hydrogen-rich water to prevent oxidative stress is limited. Respiration of hydrogen-containing air is found to be effective for Parkinson’s disease^15^, brain infarction^16^, Obesity and Diabetes^17^. However, use of this method is limited mainly because of restriction of facilities.

Although Si bulk doesn’t strongly react with water, Si nanostructures do, leading to generation of hydrogen^18–21^. Most of researches on hydrogen generation using Si nanostructures have been performed under strong alkaline conditions to achieve high hydrogen generation rates mainly for application to fuel cells. Only our research group in Osaka University has been studying hydrogen generation under the neutral pH region (i.e., pH7~9) for the medical use^22,23^. Si and the reaction product, SiO_2_, are both nonpoisonous, Si-based agent can be administrated to generate hydrogen *in vivo*.

Chronic kidney disease (CKD) is now known well as a global public health concern^24^ because there are not any procedures for improving the disease directly. In these days, it is recognized that oxidative stress has a pivotal role in developing CKD^25^ through inducing extensive tubular dilation in proximal and distal tubules, tubular debris, widening Bowman’s space^26^. In addition, CKD patients often have many underlying factors, including hypertension, diabetes mellitus, hyperlipidemia. These factors are involved in developing renal damages through oxidative stress^27–29^. Oxidative stress breaks the balance between free radicals and antioxidant capacity^24^ and contributes to activating some transcription factors. For example, TGF-β, nuclear factor-kappa B (NF-κB), and inflammatory cytokines including IL-1 β stimulate cell hypertrophy, inflammation, and up-regulation of growth factors^30–32^. These inflammatory changes under the production of OH radicals induce apoptotic cell death^33^. Therefore, it is crucial to suppress OH radicals to keep renal function better. Current therapy is mainly focused on slowing CKD progression: blood pressure control and renin–angiotensin system blockade. In other words, no methods have been developed to improve CKD completely, without the ultimate therapy: kidney transplantation. Considering the fact that the number of CKD patients continues to increase, the development of new therapeutic tools is necessary. In the present study, we have investigated the renoprotective effects of Si-based agents with 5/6-nephrectomized rats, which have remained the developed prototype closely mimics human CKD^24^.

Parkinson’s disease (PD) is a degenerative disease characterized by loss of dopamine neurons in the nigrostriatal pathway and leads to clinical symptoms such as rest tremor, bradykinesia, rigidity, and impaired balance^34^. Although the exact pathophysiology of PD is not clearly elucidated, oxidative stress plays a key role in degenerative process of dopaminergic neurons^35^. The sources of ROS are produced by dopamine metabolism, dysfunction of mitochondria and neuroinflammation in PD. Increased lipid oxidation, oxidative DNA damage, and oxidative protein damage have been studied in human postmortem brains with PD^35^. 6-hydroxydopamine (6-OHDA) is widely used toxin for degeneration of the nigrostriatal dopaminergic pathway as an animal model of PD because it closely resembles to the symptoms of human PD^35^. 6-OHDA induces protein carbonylation, an oxidative modification, and activates caspase-3^36^. On the other hand, hydrogen acts as a selective antioxidant. Hydrogen-rich water has been reported to attenuate dopaminergic neuronal damage in animal models^37^. In the present study, we have examined the neuroprotective effects of Si-based agent in a model of PD induced by 6-OHDA.

## Methods

### Si-based agent

Si-based agent was fabricated from Si powder (Koujundo Chemical Laboratory Si 3 N Powder ca. 5 µm). Si powder was milled with the beads milling method. The average diameter of Si crystallites was estimated to be 23 nm from analysis of the X-ray diffraction Si(111) peak. Surface treatment and aggregation treatment were performed on fabricated Si nanopower to produce Si-based agent. Therefore, Si-based agent mainly consisted of agglomerates of Si nanopoweder.

### Si-based agent-containing feed

For control rats, we used AIN93M (Oriental Yeast Co., Ltd., Tokyo, Japan). In addition, for our experiments, we made special feed containing 0.1wt.%, 0.5wt.%, 1.0wt.%, and 2.5wt.% Si-based agent in AIN93M, respectively. Before animal experiments, we examined the hydrogen production from this feed and water using a sensor gas chromatograph, SGHA-PA (FIS Inc., Hyogo, Japan).

### Measurements of hydrogen volume generated by the reaction with water

Si-based agent was immersed in water at 36 °C with pH in the region between 8.3 and 9.0. For adjusting pH at 8.3, sodium hydrogen carbonate (NaHCO_3_) was employed while for adjustment of pH at 8.5 and 9.0, both NaHCO_3_ and sodium carbonate (Na_2_CO_3_) were used. The generated hydrogen volume was determined by measurements of the hydrogen concentration in the solutions by use of a TOA DKK-TOA DH-35A potable dissolved hydrogen-meter.

### Animal ethics

All experiments were approved by the animal ethics committee of Osaka University and performed according to the guidelines of the National Institute of Health (NIH) Guide for Care and Use of Laboratory Animals.

### Experimental design of the rat remnant kidney model

Thirty male Sprague-Dawley rats (Japan SLC Inc., Shizuoka, Japan) weighing 180–250 g were used and were maintained under standard conditions until the experiments were done.

The rats were randomly allocated into five groups: 1) the normal feed group (control group; n = 10); and 2–5) the Si-based agent feed group (0.1wt.% Si-based agent group; n = 8, 0.5wt.% Si-based agent group; n = 6, 1.0wt.% Si-based agent group; n = 6). After starting the feed for one week, rats underwent subtotal (5/6) nephrectomy, as reported previously^38,39^. In brief, we performed by infarction of approximately two-thirds of the left kidney by selective ligation of two of 3–4 extrarenal branches of the left renal artery and resected 2/3 of the kidney mass, followed by removal of the right kidney one week later. From now on, in the text and Figures, the control group means the 5/6 nephrectomized rat. The rats were euthanized at the 8th week. At the 8th week, we collected blood and urine samples, and measured serum creatinine (mg/dl), urinary protein and creatinine for measuring urinary protein (mg/gCr). In addition, we measured urinary 8-OHdG levels for investigating oxidative DNA damage^40^ with the enzyme-linked immunosorbent assay (ELISA) at Nikken Seil Co., LTD. (Shizuoka, Japan).

### Morphological assessment for the rat remnant kidney model

At the given endpoint, tissue samples were fixed in liquid nitrogen and fixed on slides with 4% PFA. Four μm tissue sections were deparaffinized with xylene and stained with Masson’s trichrome. The diameter of glomeruli (at least 30) in each group was measured for examining the compensatory renal hypertrophy, which occurs following the progression of CKD. Moreover, the fibrotic area stained blue by Masson’s trichrome staining was measured.

Immunohistochemical staining was performed using the LSAB2 System-HRP (Dako, Hamburg, Germany), according to the manufacturer’s instructions. Endogenous peroxidase activities were blocked with Peroxidase Suppressor (Thermo Fisher Scientific, Waltham, MA) and Avidin/Biotin Blocking Kit (Vector Laboraories, Burlingame, CA) for each 15 min. The first antibodies were diluted in REAL antibody Diluent (Dako) at specific concentrations as described below and then incubated for 16 h at 4 °C. To detect myofibroblasts, we used anti-human α-SMA antibody (1:50, Dako). For the analysis of oxidative stress, 8-OHdG (1:5, Nikken SEIL, Shizuoka, Japan) immunostaining was performed. These were followed by incubation with suitable secondary antibodies. All incubations were performed in a humidified chamber. The chromogenic color was developed with Liquid DAB + Substrate Chromogen System (Dako). The nuclei were counterstained with methyl green solution (FUJIFILM Wako Pure Chemical Corp., Osaka, Japan).

All histological slides were examined by light microscopy using a KEYENCE BZ-X710 (Keyence, Osaka, Japan); pictures were taken with the analysis application (Keyence BZ-H3A). The area of endothelial cells stained with α-SMA-positive myofibroblasts relative to the total area of the field was calculated as a percentage by a computer-aided manipulator. Moreover, the injury score of glomeruli was assessed according to previously described method scored from 0 to 4 with α-SMA staining: 0, no mesangial matrix expansion; 1, minor; 2, weak; 3, moderate; and 4, strong^41^. As for 8-OHdG immunostaining, the rate of 8-OHdG positive cells was calculated. The scores of ten fields per kidney were averaged, and the mean scores for each group were then averaged.

### TUNEL staining for rat remnant kidney model

TUNEL staining was performed using the *in situ* Apoptosis Detection Kit (Takara Bio, Otsu, Japan), according to the manufacturer’s instructions as previously described^42^. Briefly, the sections were deparaffinized and treated with antigen retrieval in preheated 10 mmol/l sodium citrate (pH 7) using a steamer for 40 min. They were then incubated with 3% H_2_O_2_ for 10 min, which was followed by incubation with TdT enzyme solution for 90 min at 37 °C. The reaction was terminated by incubation in a stop/wash buffer for 30 min at 37 °C. The number of TUNEL-positive cell nuclei and the total numbers of cell nuclei stained with methyl green were counted in ten random areas, and the percentages of the numbers of TUNEL-positive nuclei to the numbers of total cell nuclei were calculated.

### Quantitative real-time reverse transcription-polymerase chain reaction for the rat remnant kidney model

Total RNA was extracted from the cortex of the treated kidney with RNeasy Plus Universal Mini Kit (Qiagen) according to the manufacturer’s directions. Aliquots (10 μl) of 500 ng of RNA were reverse transcripted to complementary DNA using PrimeScript RT reagent kit (Takara Bio) according to manufacturing protocol. Quantitative real-time reverse-transcriptase polymerase chain reaction was performed using ABI PowerUp SYBR Green Master Mix on an ABI Quant Studio 7 (Thermo Fisher Scientific). Primers amplifying the rat mRNA regions designed using the Primer Express software package v3.0.1 (Thermo Fisher Scientific, No4363991) or referred as previously published^43^. The PCR primer sets used for cDNA amplification were as follows: caspase-3 sense 5- AAT TCA AGG GAC GGG TCA TG -3, antisense 5- GCT TGT GCG CGT ACA GTT TC -3; CCL2 sense 5-CTA TGC AGG TCT CTG TCA CGC TTC-3, antisense 5-CAG CCG ACT CAT TGG GAT CA-3; IL-6 sense 5-ATT GTA TGA ACA GCG ATG ATG CAC-3, antisense 5-CCA GGT AGA AAC GGA ACT CCA GA-3; TIMP-1 sense 5-CGA GAC CAC CTT ATA CCA GCG TTA-3, antisense 5-TGATGTGCAAATTTCCGTTCC-3; β-actin 5-GGA GAT TAC TGC CCT GGC TCC TA-3, antisense 5-GAC TCA TCG TAC TCC TGC TTG CTG-3. Data are expressed as the comparative cycle threshold. The normalized cycle threshold value of each gene was obtained by subtracting the cycle threshold value of β-actin RNA.

### Statistical analysis for chronic kidney disease model

The Prism software program was used for the statistical analysis (Prism version 7.0; Graph Pad Software, La Jolla, CA). All results are given as the means ± standard deviation. Statistical analyses were performed using the Tukey test for parametric multiple comparisons. P values less than 0.05 were considered to be significant.

### Mouse models of 6-OHDA-induced Parkinson’s disease

All mice were fed AIN93M (Oriental Yeast Co., Ltd., Tokyo, Japan) in a week before brain lesion. Taking into account allometric scaling theory^44^, we gave mice a higher dose of Si-based agent than rats. In addition, Si-based agent generates hydrogen mainly in the intestine. The hydrogen generated in the intestine can spread throughout the body. Since the brain is farther from the intestine than the kidney, we have used a higher concentration of Si-based agent-containing diets (2.5%) to provide the protective effects on the brain. Mice were anesthetized with three-in-one admixture solution (0.3 mg/kg of medetomidine, 4.0 mg/kg of midazolam and 5.0 mg/kg of butorphanol in 0.9% saline; intraperitoneal injection)^45^. After fixing in stereotaxic apparatus (Muromachi Kikai Co., Tokyo, Japan), mice were injured with a single infusion of 6-OHDA (Sigma-Aldrich, St. Louis, MO, USA) as mentioned below. Three µl dopaminergic neurotoxin solution, saline including 0.2% 6-OHDA and 0.2% ascorbic acid (FUJIFILM Wako Pure Chemical Co., Osaka, Japan) were stereotactically microinjected into the left striatum using 10 µl Hamilton syringe (Sigma-Aldrich) at the following coordinates; A-P: 0.5 mm, M-L: −2.4 mm, D-V: −3.1 mm in mm with respect to bregma^46,47^. The infusion rate was 1 µl/min for 3 min with the help auto-injector (Muromachi Kikai Co.). We allowed a few minutes for prevention of leakage of the solution before removal of needle. After unilateral brain injury, 6-OHDA-injected mice were assigned to two groups; the 6-OHDA-treated group was fed AIN93M, and the 6-OHDA + Si-based agent-treated group was fed AIN93M containing 2.5% Si-based agent (Oriental Yeast Co., Ltd.). Three behavioral tests were performed at the 4th week of 6-OHDA lesion. At the 5th week after surgery, apomorphine-induced rotary test was conducted and then all the mice were perfused for immunofluorescence staining. In the present experiments, we prepared 7 mice in the control group and 8 mice in the Si-based agent group. For all behavioral tests, we also prepared non-injected mice treated with control diets or Si-based agent diets for four weeks (6 mice per group).

### Apomorphine-induced rotation test

Apomorphine-induced rotation test was modified^48^. Four weeks post-6-OHDA lesion animals were placed in 1000 ml standard laboratory beakers with a diameter of 9 cm for 20 min. Subsequently, mice were intraperitoneally injected with 1 mg/kg apomorphine (Sigma, dopamine receptor D1/D2 agonist) in saline and placed back into the beaker. Four weeks after 6-OHDA injection into the left striatum, mice made an unaffected side turn (clockwise turn) because of denervation hypersensitivity. The number of clockwise turn in 10 min was counted.

### Behavioral tests

#### Rotarod test

Fore- and hind-limb motor coordination and balance were evaluated by an accelerating Rotarod apparatus (MK-610A; Muromachi Kikai Co.), as previously described^49^. Mice were placed on a rod (3 cm in diameter) with constant low-speed rotation (4 rpm, 30 s) to get used to walking on the rod. Then the rotation test was started at 4 rpm and gradually accelerated to 30 rpm at 5 min. The period of remaining on the rod was measured automatically. In one set of experiments, two trials per a mouse were performed. Statistical comparison among groups was conducted using the average of two trials.

#### Assessment of spontaneous motor activity using supermex system

Spontaneous motor activity of mice was individually monitored in a home cage by means of a Supermex and a photocell beam system (Muromachi Kikai Co.), as previously described^49–51^. The sensor of this system is equipped with paired infrared pyroelectric detectors and mounted in the center of a chamber’s ceiling. The radiated body heat of each mouse was detected by the sensor. The total spontaneous motor activity of mice were measured for 10 min, digitally converted, and stored. The data were analyzed by data accumulation software (CompACT AMS Ver.3: Muromachi Kikai Co.).

#### Open field test

The experimental setup has been previously described^52^. A mouse was placed in the center of the open-field apparatus (50 × 50 × 40 cm; Muromachi Kikai Co.). The total travel distance, the average speed and the activity or inactivity time during the tests were recorded using an ANY-maze video-tracking system (Stoelting Co., Wood Dale, IL, USA). Data were recorded for 10 min.

### Immunohistochemical staining for tyrosine hydroxylase

Perfusion with 4% paraformaldehyde in 0.1 M phosphate buffer (PB) was carried out after 5 weeks of surgery. The removed brains were post-fixed in the same fixative at 4 °C overnight and then cryoprotected with 30% sucrose-containing 0.1 M PB at 4 °C followed by frozen in dry-ice. The frozen brains were sectioned into 30-µm-thick. The sections were stocked in 0.01 M phosphate buffered saline (PBS) at 4 °C until use.

Immunofluorescence staining using the free-floating sections were performed as previously described^53^. The sections were rinsed in 0.01 M PBS and then immersed into 0.01 M PBS containing 0.3% Triton-X and 3% bovine serum albumin at 22 ± 2 °C for 1 h because of increase of permeability to antibody and blocking of non-specific reaction followed by treatment with rabbit anti-Tyrosine hydroxylase polyclonal antibody (1:1000; Catalog no. ab112; Abcam Cambridge, UK) at 4 °C overnight. The sections were washed for several times and then incubated at 22 ± 2 °C for 1 h with donkey anti-rabbit immunoglobulin G antibody conjugated Alexa Fluore 488 (1:500; Catalog no. A-21206; Thermo Fisher Scientific). After washing thoroughly, the samples were mounted on the microscope slides with PermaFluor (Thermo Fisher Scientific). The analysis of stained slides was performed with an Olympus microscope (BX53; Olympus Corporation, Tokyo, Japan) and a Keyence microscope (BZ-X700; Keyence).

### Statistical analysis for Parkinson’s disease model

As for all experiments, Student’s *t*-test was used to compare the differences among four groups, Control, Si-based agent, 6-OHDA, and 6-OHDA + Si-based agent groups. The results of statistical tests were considered significant at *p < 0.05 and *p < 0.01. Data are expressed as mean values ± standard error of the mean (SEM).

## Results

### Hydrogen generation from Si-based agent by reaction with water

**Figure 1:**
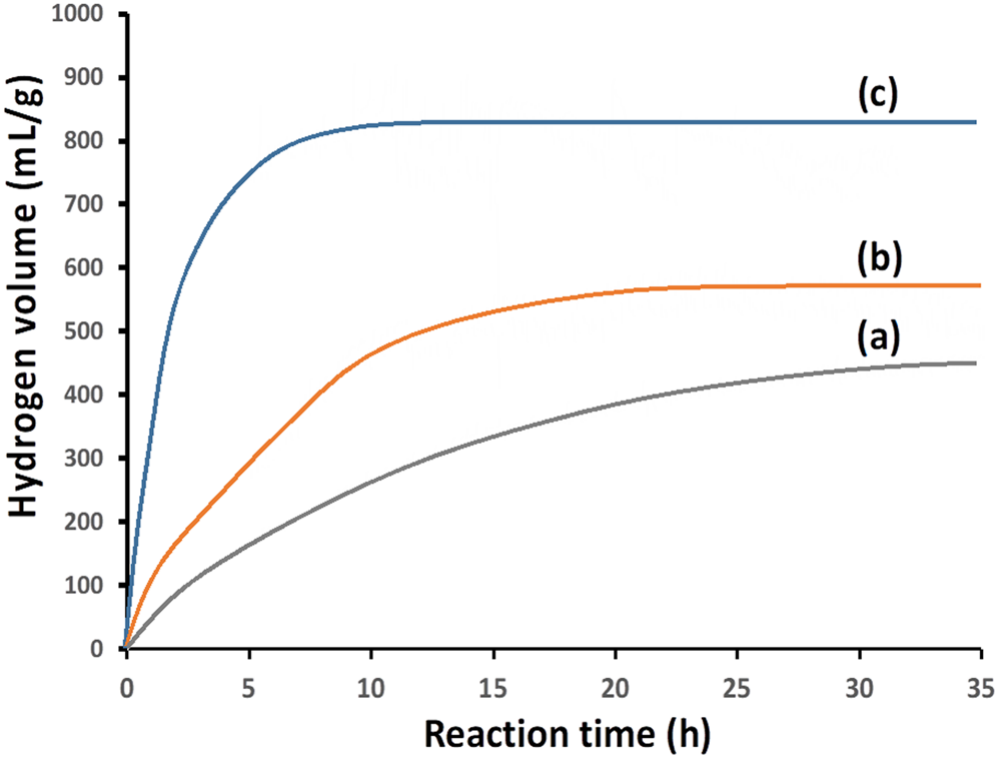
Hydrogen volume generated by the reaction of Si-based agent with water at 36 °C. (**a**) pH of the solution is adjusted at 8.3 using NaHCO_3_. (**b**,**c**) pHs of the solutions are adjusted at 8.5 and 9.0, respectively, using NaHCO_3_ plus Na_2_CO_3_. Si-based agent is composed of Si fine particles covered with silicon oxide which includes high amounts of suboxide species (cf. supplementary Fig. 3), i.e., Si atoms bound to one, two, and three oxygen atoms, each. Si fine particles aggregate to the sizes larger than 0.1 μm.

In environment similar to that in bowels, i.e., pH 8.3 and 36 °C, Si-based agent easily reacted with water, generating a high amount of hydrogen (Fig. 1a). ~400 mL hydrogen was generated in 24 h from 1 g Si-based agent. This hydrogen amount corresponds to that contained in 22 L saturated hydrogen-rich water. Moreover, the reaction for hydrogen generation proceeded for more than 24 h. Therefore, it can be concluded that developed Si-based agent satisfies the following three requirements to prevent oxidative stress-induced diseases without side-effects: i) presence of a high amount of reducing agent in the body, ii) continuous presence of reducing agent in the body, and iii) reducing agent which eliminates only OH radicals among ROS. A high amount of reducing agent is necessary in order to react with OH radicals before they oxidize cells (requirement i). Because OH radicals are continuously generated due to e.g., mitochondrial respiratory metabolism, it is required that reducing agent is always present in the body to efficiently eliminate OH radicals (requirement ii). Hydrogen reacts only with OH radicals among ROS (requirement iii).

Even in the case of a slight pH increase from 8.3 to 8.5 (cf. Fig. 1a,b), the hydrogen generation reaction was enhanced and ~500 mL/g hydrogen was generated in 12 h. Under conditions with pH 9.0 and 36 °C (Fig. 1c), ~800 mL/g hydrogen was generated in 7 h.

The reaction rate prominently increased with pH, but no hydrogen generation was observed when pH of the solutions was below 5. Therefore, when Si-based agent is administrated, the hydrogen generation reaction doesn’t proceed in a stomach where the environment is strong acidic due to gastric acid of pH~1.5 while it occurs in bowels because of the alkaline condition due to pancreatic juice of pH ~8.3. Generated hydrogen is efficiently absorbed in bowels, circulate throughout the body, and eliminate OH radicals, resulting in prevention of oxidative stress-induced diseases as described below.

Although the rate of hydrogen generation prominently increased with pH as described above, pH of the solutions didn’t change after the reaction. Considering these results, the most likely reaction scheme is written as^22^

1 $$Si+2O{H}^{-}\to Si{O}_{2}+2H(or\,{H}_{2})+e,$$

2 $$2{H}_{2}O+2e\to 2O{H}^{-}+2H(or\,{H}_{2}).$$

The reaction rate for reaction (1) is much lower than that for reaction (2), and thus, the total reaction rate strongly depends on the concentration of OH^−^ ions, i.e., pH. Reaction (1) consumes OH^−^ ions while reaction (2) generates them, and thus, after the below-described overall reaction, i.e., reaction (1) + reaction (2) = reaction (3), the concentration of OH^−^ ions, i.e., pH, doesn’t change:

3 $$Si+2{H}_{2}O\to Si{O}_{2}+4H(or\,2{H}_{2}).$$

In the *in-vitro* experiments, hydrogen molecules are generated in reactions (1) and (2) (or reaction (3)). In the body, on the other hand, it is not clear whether hydrogen molecules are generated, or generated hydrogen atoms or electrons are captured by e.g., protein containing metallic elements, followed by transfer to organs. We suppose that the latter is more probable.

Reaction (1) proceeds at the silicon oxide/Si interface, and generated electrons transfer to the surface of the silicon oxide layer, leading to reaction (2) (cf. supplementary Fig. 1). We found that after the hydrogen generation reaction under pH 8.3 at 36 °C for 24 h, a 6.8 nm thick silicon oxide layer is formed on Si-based agent (cf. supplementary Figs. 2 and 3).

### Renoprotective effects of Si-based agent on remnant kidney disease rat

**Figure 2:**
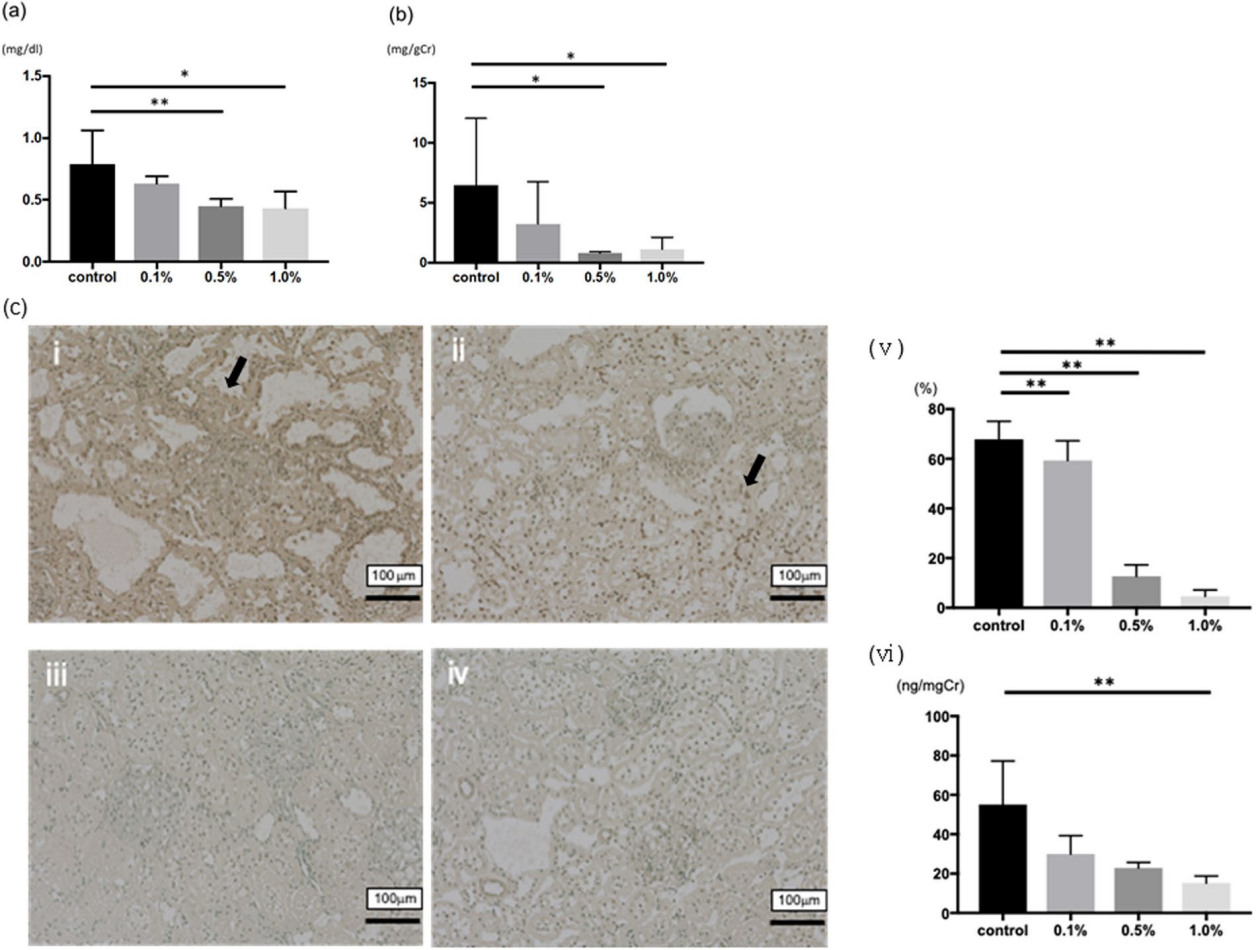
The effects of Si-based agent on renal function and the impacts of oxidative stress. (**a**) The effects of Si-based agent on serum creatinine. (**b**) The effects of Si-based agent on urinary protein. (**c**) 8-hydroxydeoxyguanosine (8-OHdG) staining after for measuring the impact of oxidative stress. The dark brown dots (arrow) correspond to representative 8-OHdG-positive nuclei in the control group (i) or the Si-based agent (0.1%–1.0%) groups (ii-iv). Scale bar: 100 µm. We counted the number of 8-OHdG-positive cells per 100 cells in ten random areas of each rats (v). In addition, we measured the urinary 8-OHdG level with ELISA kit (vi). Data were expressed as mean +/− SD. **p < 0.01, *p < 0.05 vs. the control group, respectively.

The effects (serum creatinine and urinary protein) of Si-based agent diet in 5/6-nephrectomized model rats are summarized (Fig. 2a,b). Compared to the control group (0.79 ± 0.27 mg/dl), 0.5wt.% and 1.0wt.% Si-based agent diet significantly suppressed the increase of serum creatinine (0.45 ± 0.06 mg/dl and 0.43 ± 0.14 mg/dl, respectively). In addition, the level of urinary protein significantly suppressed by Si-based agent diet (0.80 ± 0.12 mg/gCr in 0.5wt.% group and 1.12 ± 1.00 mg/gCr in 1.0wt.% group, respectively) compared to the control group (6.49 ± 5.55 mg/gCr).

For measuring oxidative stress in the 8th week, we examined the number of 8-hydroxydeoxyguanosine (8-OHdG) positive cells in the kidney tissues and urinary 8-OHdG levels of each group. The number of positive cells was significantly suppressed in the Si-based agent diet groups (Fig. 2ci-v). Besides, urinary 8-OHdG levels are significantly suppressed in 1.0wt.% Si-based agent diet group (15.2 ± 3.6 ng/mgCr) comparing with the control group (55.1 ± 22.1 ng/mgCr, Fig. 2cvi).

**Figure 3:**
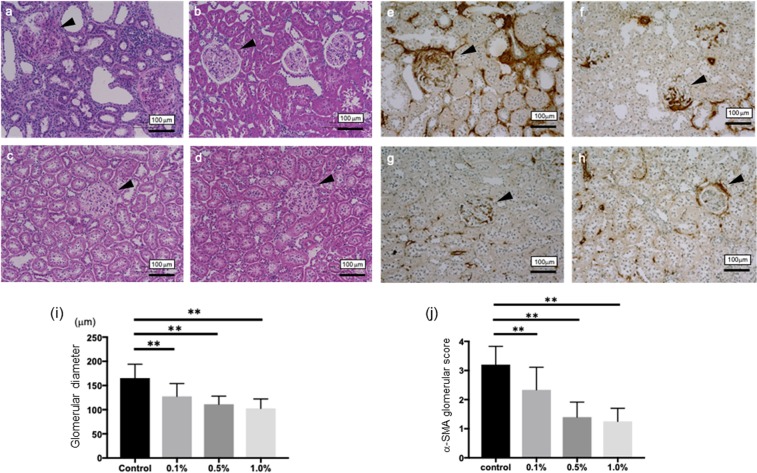
Representative morphological changes in the control group (**a,e**) or the Si-based agent (0.1wt.%: b,f, 0.5wt.%: c,g, 1.0wt.%: **d,h**) groups, respectively. Scale bar: 100 µm. The dilations of glomeruli and the increase of mesangial matrix (arrowhead) are examined. The diameters of glomeruli were assessed using a color image analyzer and compared among each group (i). Mesangial matrix levels were assessed according to previously described method scored from 0 to 4 with α-SMA staining: 0, no mesangial matrix expansion; 1, minor; 2, weak; 3, moderate; and 4. The scores were averaged and compared among each group (j). (**p < 0.01, *p < 0.05).

**Figure 4:**
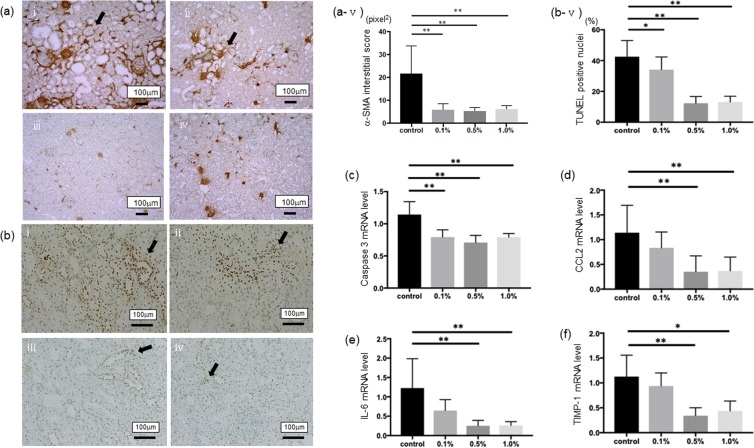
The anti-apoptotic and anti-inflammatory effects of Si-based agent on renal function. (**a**) Interstitial phenotypic changes are assessed by immunohistochemical staining of α-SMA (arrow) in the control group (i) or the Si-based agent (0.1wt.%-1.0wt.%) groups (ii-iv). The positive area was quantitatively assessed using a color image analyzer (v). (**b**) TUNEL method was performed for measuring the levels of apoptosis in each group as describe above (i-iv). We counted the number of TUNEL-positive cells (arrow) per 100 cells in ten random areas of each rats (v). Scale bar: 100 µm. (**c-f**) The normalized messenger RNA (mRNA) level of caspase-3 (**c**), C-C motif chemokine ligand 2 (CCL2) (**d**), interleukin-6 (IL-6) (**e**), and a tissue inhibitor of metalloproteinases (TIMP-1, **f**) were measured. The levels are normalized to β-actin levels for each sample. The y-axis values represent the number of copies relative the number of copies in the same samples. The levels are normalized to β-actin levels for each sample. The y-axis values represent the number of copies relative the number of copies in the same samples. Data were expressed as mean +/− SD. **p < 0.01, *p < 0.05 vs. the control group, respectively.

To investigate the compensatory renal hypertrophy, which occurs following the progression of CKD, we measured the diameters of glomeruli in each group. Comparing to the control group, the sizes of glomeruli in Si-based agent diet groups are significantly small (Fig. 3a-d,i). The interstitial fibrotic area at the 8th week was stained on Masson’s trichrome staining (Supplementary Fig. 4a-d). The control group showed the progression of interstitial fibrosis. In parallel with the finding, Si-based agent diet administration significantly suppressed the development of interstitial fibrosis (Supplementary Fig. 4e). To detect interstitial myofibroblasts, which were associated with damage and fibrosis, the expression of α-smooth muscle actin (α-SMA) was examined immunohistochemically. In addition, we measured the mesangial matrix levels of glomeruli in each group to investigate the progression of CKD. The expression increased in the 8th week in the control group (Fig. 4ai,v), whereas Si-based agent diet significantly suppressed interstitial expression of α-SMA (p < 0.01 vs. the control group, Fig. 4aii-iv). Especially in 0.5wt.% and 1.0wt.% Si-based agent diet groups, the expression of α-SMA was limited to the blood vessels. On the other hand, the progression of mesangial matrix level was suppressed in each Si-based agent diet group (p < 0.01 vs. the control group, Fig. 3e-h,j).

To investigate whether the Si administration could provide protection for tubular epithelial cells against apoptosis, the apoptotic bodies, labeled as an *in situ* end-labeled DNA fragment with the terminal deoxynucleotidyltransferase-mediated dUTP nick end-labeling (TUNEL) method, were examined (Fig. 4b). Tubular apoptosis was persistently increased after nephrectomy in parallel with the tubular damage (control group, 42.5 ± 10.5 cells per 100 cells, Fig. 4bi), whereas the administration of Si-based agent diet (34.1 ± 8.23 cells on 0.1wt.% group; P < 0.01 vs. the control group, 12.3 ± 4.40 cells on 0.5wt.% group and 13.1 ± 3.78 cells on 1.0wt.% group; P < 0.01 vs. the control group, respectively, Fig. 4bii-v) inhibited tubular apoptosis. As the previous report, it is well known that cleaved caspase-3-positive cells are histologically increased for apoptosis in 5/6-nephrectomized kidney model^24^. We investigated the messenger RNA (mRNA) level of caspase-3. Compared to the control group, the mRNA was significantly decreased in the Si-based agent diet groups (p < 0.01, Fig. 4c).

We measured C-C motif chemokine ligand 2 (CCL2) and interleukin-6 (IL-6) to investigate the metabolic pathway. CCL2, which was a chemokine and recruits memory T cells and monocyte to the sites of inflammation, and IL-6 which was a pro-inflammatory cytokine in the kidney tissues, were significantly suppressed by the 0.5wt.% and 1.0wt.% Si-based agent diet groups in comparison to the control group (Fig. 4d,e). Moreover, we examined the mRNA level of a tissue inhibitor of metalloproteinases (TIMP-1), which was a member of the TIMP family and was one of the biomarkers of interstitial fibrosis. Compared to the control group, the mRNA was significantly decreased in the 0.5wt.% and 1.0wt.% Si-based agent diet groups (Fig. 4f).

Taken together, these results indicate that the treatment with Si-based agent is useful for inhibiting oxidative stress injury and renal damages through suppressing inflammation and apoptosis. Long-term continuous therapy with hydrogen generated by Si-based agent can be considered as epochal therapy against kidney damage in chronic kidney disease.

### Neuroprotective effects of Si-based agent on 6-OHDA-induced Parkinson’s disease mice

**Figure 5:**
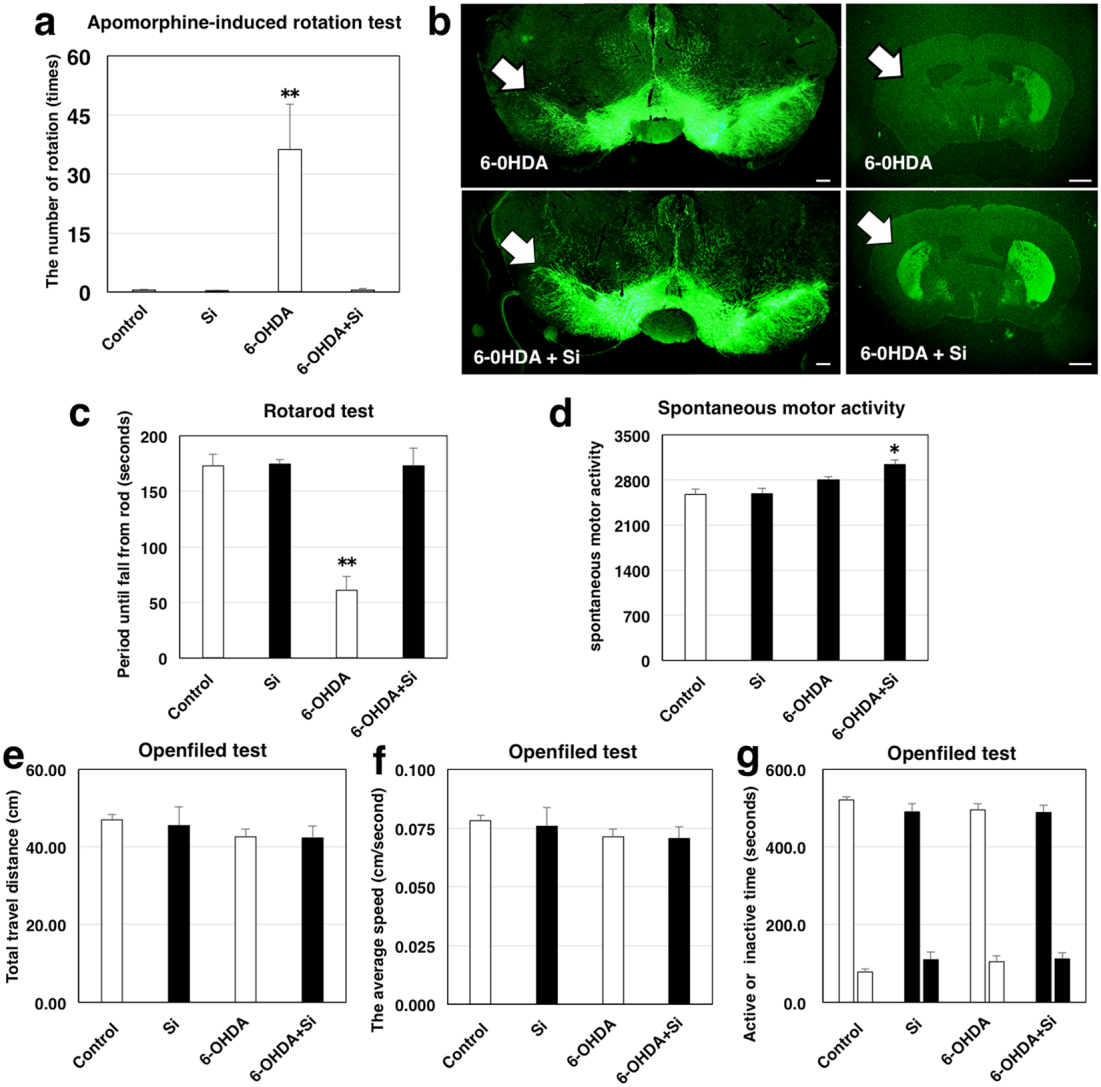
Neuroprotective effects of Si-based agent in 6-OHDA-induced hemiparkinsonian mice. (**a**) The histogram of the number of an unaffected side turn (right hand turn) in apomorphine-induced rotation test. The control group (white bar, first from left), the Si-based agent group (black bar, second from left), the 6-OHDA group (white bar, third from left) and the 6-OHDA + Si-based agent group (black bar, fourth from left). Data were expressed as means ± SEM of six (Control group), six (Si-based agent group), seven (6-OHDA group) or eight animals (6-OHDA + Si-based agent group). **P < 0.01 vs. the 6-OHDA group (Student’s t-test). (**b**) The micrographs of tyrosine hydroxylase staining for substantia nigra pars compacta (left side) and striatum (right side). White arrow indicated lesion site. Opposite side was contralateral control. Upper images: the 6-OHDA group. Lower images: 6-OHDA and silicon groups. Scale bar: 200 µm (left) and 1 mm (right). (**c**) The histogram of the time that mice stayed on a rotating rod in rotarod test. (**d**) The histogram of the spontaneous motor activity measured by Supermex system. (**e–g**) The histograms of the following parameters evaluated by Open field test: total travel distance (**e**), the average speed (**f**) and Active or inactive time (**g**). Control group (white bar, first from left), silicon group (black bar, second from left), 6-OHDA group (white bar, third from left) and 6-OHDA + silicon group (black bar, fourth from left). All data were expressed as means ± SEM of six (Control group), six (Si-based agent group), seven (6-OHDA group) or eight animals (6-OHDA + Si-based agent group). *P < 0.05 or **P < 0.01 vs. 6-OHDA group (Student’s t-test).

In order to estimate the effect of Si-based agent on a central nervous system (CNS) disease, we performed *in vivo* studies using an unilateral 6-OHDA lesion mouse model of PD. The PD mouse model was divided into two groups, 6-OHDA-treated mice fed with control diet (6-OHDA group) and 6-OHDA-treated mice fed with Si-based agent-containing diet (6-OHDA + Si-based agent) group. For comparison, mice without 6-OHDA injection fed with control diets (control group) and 2.5wt.% Si-based agent-containing diets (Si-based agent group) were prepared. 6-OHDA is known to induce degeneration of the dopamine neurons in the substantia nigra pars compacta (SNpc). Therefore, 5 weeks after surgery, we performed apomorphine-induced rotation test to estimate dopaminergic neuronal loss. Apomorphine caused mice to rotate in a direction contralateral side of the lesion (clockwise direction) in the 6-OHDA group, whereas almost no rotation was observed in the 6-OHDA + Si-based agent group as in the non-injected groups (in Fig. 5a, 0.5 ± 0.2 for the control group, 0.3 ± 0.2 for the Si-based agent group, 36.3 ± 11.4 for the 6-OHDA group, and 0.5 ± 0.3 for 6-OHDA + Si-based agent group). Subsequently, we also morphologically examined the effect on the dopaminergic neurons by immunohistochemistry with antibody against tyrosine hydroxylase as a marker for dopamine neurons. Dopaminergic cell bodies and dendrites were localized in the SNpc, whereas dopaminergic nerve terminals were seen in the striatum (Str). In both the 6-OHDA and 6-OHDA + Si-based agent groups, dopaminergic neurons of the left SNpc (ipsilateral to lesion site) showed fewer neurites and soma than those of the right SNpc (contralateral) (Fig. 5b left, Supplementary Fig. 5a,c,e,g). However, the dopaminergic neurons of the 6-OHDA group received much more damage than those of the 6-OHDA + Si-based agent group. Moreover, although the TH positive dopaminergic terminals of the ipsilateral Str were dramatically decreased in the 6-OHDA group, a number of surviving dopaminergic terminals of the ipsilateral Str were observed in most of the 6-OHDA + Si-based agent group (Fig. 5b right, Supplementary Fig. 5b,d,f,h). Both groups of mice equally displayed TH positive immunoreactivities in the contralateral Str. These findings demonstrated that Si-based agent significantly attenuated degeneration of dopaminergic neurons caused by 6-OHDA.

Subsequently, we evaluated the motor balance and coordination using the rotarod test because unilateral lesion mouse model of PD exhibited impairment of ability in the motor balance and coordination^54^. The retention time in the rotarod test of the 6-OHDA + Si-based agent group was significantly longer than that of the 6-OHDA group and not significantly different from that of non-injected groups (in Fig. 5c, 172.8 ± 10.7 for the control group, 174.5 ± 4.2 for the Si-based agent group, 60.8 ± 13.0 for the 6-OHDA group, and 173 ± 16.0 for the 6-OHDA + Si-based agent group). Hence, the 6-OHDA + Si-based agent group maintained motor coordination performance despite dopaminergic toxicity induced by 6-OHDA. In addition, although there was a statistical difference in spontaneous motor activity between the 6-OHDA + Si-based agent group and other groups, the difference is small when comparing the average value (in Fig. 5d, 2834.5 ± 41.1 for the control group, 2885.5 ± 21.8 for the Si-based agent group, 2807 ± 44.7 for the 6-OHDA control group, and 3035 ± 80.1 for the 6-OHDA + Si-based agent group). There were no significant differences in the numerical data of each parameter (total travel distance, the average speed and activity time or inactivity time) assessed by Open field test (Fig. 5e-g control group, Si-based agent group, 6-OHDA group and 6-OHDA + Si-based agent group: total travel distance 47.0 ± 1.4, 45.5 ± 4.8, 42.7 ± 1.9, and 42.3 ± 3.0, the average speed 0.078 ± 0.002, 0.076 ± 0.008, 0.071 ± 0.003, and 0.071 ± 0.005, activity time 521.8 ± 8.1, 490.4 ± 20.5, 495.5 ± 16.0, and 489.0 ± 17.6 and inactivity time 78.2 ± 8.1, 109.6 ± 20.5, 104.5 ± 16.0, and 110.9 ± 17.6, respectively). Unlike motor balance and condition, there was no remarkable difference in motor activity between the Si-based agent and the control groups.

## Discussion

Si-based agent can generate a high amount of hydrogen (i.e., ~400 mL/g) in simulated conditions in bowels (i.e., pH 8.3 and 36 °C) for more than 24 h, and therefore, it can effectively decrease oxidative stress, leading to prevention of diseases caused mainly by oxidative stress. The hydrogen generation reaction rate prominently increases with pH, and the reaction doesn’t occur under acidic environments. Therefore, by taking Si-based agent, the hydrogen generation reaction doesn’t proceed in stomach while it does in bowels because of alkaline environment due to pancreatic juice.

Only hydrogen generated from Si-based agent is absorbed in the body, while Si-based agent is hardly absorbed, and therefore, Si-based agent is thought to be physiologically highly safe. In fact, by 91 days repeated oral toxicity test, and no death example was observed, and no abnormality was seen in a general state and autopsy either. No abnormality was observed in the MTT assay either. By chromosome aberration test and reverse mutation test, no abnormality was observed either.

In the present study, we assessed the renoprotective effect of Si-based agent through suppressing inflammation and apoptosis with 5/6 nephrectomized model rats. Oxidative stress increases with deteriorating renal function through them from the early CKD stage^55^, and finally develops kidney into end-stage disease. For long time, many authors investigate the efficacy of hydrogen-rich water against oxidative stress, but it is difficult to keep high level hydrogen concentration because of the rapid diffusion. Therefore, Si-based agent is worth using for CKD because it produces a high amount of hydrogen molecules continuously. The overexpression of extracellular matrix in glomeruli and mesangial cells, proximal and distal tubules were dramatically suppressed by Si-based agent in the experiments we performed. As decreasing renal function, hyperfiltration of glomerulus is induced, possibly as a result of an early increase of renal blood flow. This would then increase the work of the glomeruli and tubules, which in turn would result first in cellular hypertrophy. For preventing the development of CKD, we use anti-aldosterone agents (e.g., angiotensin receptor blockers) in clinical field for protecting glomeruli through decreasing renal blood pressure and blood flow. Moreover, we use the agents for suppressing aldosterone which promotes the generation of ROS and activates the epidermal growth factor receptor, resulting in cell proliferation and fibrosis^56^. But, in fact, it is difficult to inhibit the development of renal failure in most cases with the administration of these medicines only. Si-based agent which can generates a high amount of hydrogen molecules might be helpful to avoid the risk, falling into end-stage kidney disease.

Oxidative stress contributes to falling into several diseases that predispose to CKD, such as hypertension, diabetes, and atherosclerosis promoting directly and indirectly the progression of renal damage^57^. Moreover, oxidative stress has a pivotal role in both the acceleration of glomerular filtration rate (GFR) deterioration and the progression of cardiovascular diseases (CVD) through atherosclerosis^58^. The Si-based agent might be able to decrease the incident rates of these diseases with hydrogen.

In this study, we used he Si-based agent for one week before performing 5/6 nephrectomy and received excellent results, suppressing the development of CKD. Unfortunately, in the clinical field, we have not established the methods for complete treatment against CKD yet. It is already investigated that a low dose of Si is harmless for humans. Therefore, we might be able to treat CKD, especially for early-stage patients. In addition, we can speculate the efficacy of the Si-based agent for dialysis patients. The most common disease that can’t be cured and cause the death of dialysis patients is CVD. Considering the efficacy for CVD, Si-based agent may be able to improve the mortality of dialysis patients. Given our results, the clinical use of Si-based agents for CKD patients should be considered as a means to decreasing mortality. Thus, further investigation, including clinical trials, is needed to establish the feasibility and efficacy of using Si-based agent.

Our study also revealed that Si-based agent prevented nigrostriatal dopaminergic neurodegeneration and exerted protective effects on motor balance and coordination in the PD model induced by 6-OHDA. Differences in motor activity were not detected between 6-OHDA-injected mice and control mice in the open field test and Supermex system in the present study. The reason why we could not detect significant differences between them was because the contralateral dopamine neurons were intact and the ipsilateral dopaminergic degeneration is partial in this hemi-parkinsonism model. In addition, lateral imbalance is not directly reflected in the measurements of the motor activity. On the other hand, the apomorphine test and rotarod test, which can recognize slight differences between affected and unaffected sides, were able to detect lateral imbalance of dopaminergic activity in the hemi-parkinsonism model unilaterally injected with 6-OHDA.

Various factors such as oxidative stress, mitochondrial dysfunction, abnormal protein aggregation, neuroinflammation, and autophagy impairment are involved in the pathogenesis of PD. Above all, mitochondrial dysfunction is closely associated with oxidative stress^35^. Parkin, PINK1 (PTEN-induced kinase 1), and DJ-1 are proteins that maintain ROS homeostasis in mitochondria, and mutations in these genes cause autosomal recessive early-onset PD^59,60^. Oxidative stress can also affect the toxicity of α-synuclein that is strongly associated with PD and, conversely, aggregation of α-synuclein enhances oxidative stress^61^. 6-OHDA is selectively up-taken into dopamine neurons by dopamine transporters, inhibits mitochondrial complex I, and generates high levels of ROS, resulting in progressive dopaminergic neuronal death^62^. Our results revealed that Si-based agent prevented nigrostriatal dopaminergic neurodegeneration induced by 6-OHDA. Our findings can be explained by the mechanism that Si-based agent generates a high amount of hydrogen molecules continuously, which results in scavenging oxidative stress in the mouse PD model induced by 6-OHDA.

There is no treatment that can stop the progression of PD. Most of the drugs have focused on treating the symptoms of PD. There is an urgent need to develop new drugs for PD. Randomized double-blind study demonstrated that oral intake of hydrogen water for 48 weeks led to a significant improvement in the total Unified Parkinson’s Disease Rating Scale (UPDRS) score in levodopa-medicated PD patients^15^, whereas no significant difference in the total UPDRS score were found between hydrogen water group and placebo water group in a larger-scale longer-term study^63^. The results of these clinical trials suggest that hydrogen may be a potential therapeutic drug for PD by developing a new method of administration. Thus, Si based agent which can generate higher amount of hydrogen in the intestine for more than 24 hours may be a promising candidate drug for PD.

In the present study, we have developed Si-based agent that generates a large amount of hydrogen continuously. The Si-based agent showed renoprotective and neuroprotective effects in animal models. Medical application of the Si-based agent can be an innovative approach to treating a wide variety of diseases mediated by oxidative stress.

## Supplementary information

Supplementary Information.
