# Supplementary material for: Renoprotective and neuroprotective effects of enteric hydrogen generation from Si-based agent
Source: Sci Rep. 2020 Apr 3;10:5859. doi: 10.1038/s41598-020-62755-9 (PMC7125117; doi:10.1038/s41598-020-62755-9)
Supplement: Supplementary file 1 — Supplementary Information. [file 41598_2020_62755_MOESM1_ESM.docx]

SUPPLEMENTARY INFORMATION

Renoprotective and neuroprotective effects of enteric hydrogen generation from Si-based agent

Yuki Kobayashi, Ryoichi Imamura, Yoshihisa Koyama, Makoto Kondo, Hikaru Kobayashi, Norio Nonomura, and Shoichi Shimada

**Supplementary Fig. 1** shows the reaction model of Si-based agent. OH^－^ ions in an alkaline solution, e.g., pH 8.3 solution, are adsorbed on the silicon oxide surface, and OH^－^ ions migrate through silicon oxide (step 1), which migration is promoted by the electrical field generated by adsorbed OH^－^ ions^1^. The migration of OH^－^ ions through silicon oxide has been found to be the rate-determining step^2^. Then, the interfacial reaction (i.e., reaction (1)) proceeds at the silicon oxide/Si-based agent interface (step 2). Generated electrons move outward through the silicon oxide layer by tunneling or by conducting in the conduction band of silicon oxide (step 3), and the surface reaction (i.e., reaction (2)) proceeds at the silicon oxide surface (step 4).

**Supplementary Fig. 2** shows the XPS spectra in the Si 2p region for Si-based agent observed before and after reaction with pH 8.3 NaHCO_3_ aqueous solutions at 36 ºC for various periods. The peak at 97.5 eV was attributable to Si and the broader peak in the higher energy region was due to silicon oxide^3,4^. The spectra were deconvoluted by the following method: The background was removed using the Shirley method^5^. Considering Si 2p_3/2_ and 2p_1/2_ components with the intensity ratio of 2:1 separated by 0.61 eV^6^, the spectra were resolved into five sets of the doublet peaks due to Si^0^, Si^+^, Si^2+^, Si^3+^, and Si^4+^ (i.e., Si atoms bound to zero, one, two, three, and four oxygen atoms each, respectively)^3,4^. Si^0^, Si^+^, Si^2+^, Si^3+^, and Si^4+^ correspond to Si_2_O, SiO, Si_2_O_3_, and SiO_2_, respectively. In Supplementary Fig. 2, only the Si 2p_3/2_ components are displayed.

The intensity of the Si^0^ peak decreased with the reaction time while that of the silicon oxide peak increased with it, indicating an increase in the silicon oxide thickness with the reaction time. After the hydrogen generation reaction for 24 h, the peak due to silicon oxide became very strong while the intensity of the peak due to Si^0^ greatly decreased, and the oxide thickness was estimated to be 6.8 nm. It should be noted that in the case of thermal oxidation of Si wafers, 6.8 nm silicon oxide is formed only at high temperatures above 700 °C^7^. In addition to the electrical field induced in the silicon oxide layer by adsorption of OH^－^ ions, inner migration of OH^－^ ions may be promoted by high concentration suboxide species (i.e., Si_2_O, SiO, and Si_2_O_3_), because suboxide species possess energy levels in silicon oxide band-gap and OH^－^ ions transfer across silicon oxide by hopping the gap-states.

**Supplementary Fig. 3** shows the thickness of SiO_2_ and suboxide species vs. the reaction time. The thickness of a silicon oxide layer and the amount of suboxide species can be estimated from analysis of the XPS spectra with the following procedure: For estimation of the thicknesses of the silicon oxide layer, the concentration of suboxide species is assumed to be uniform in the silicon oxide layer. For Si-based agent, cylindrical shape with the height the same as the radius is assumed for Si-based agent. In this case, the ratio in the intensity of the peak due to all the oxide species, $I_{ox}$, to that of Si^0^, $I_{Si}$, is given by^8^

$\frac{I_{ox}}{I_{Si}}=\frac{N_{ox}\sigma_{ox}\lambda_{ox}}{N_{Si}\sigma_{Si}\lambda_{Si}}\frac{{(R-t_{ox})}^{2}\left[ 1-\exp\left( \frac{t_{ox}}{\lambda_{ox}} \right) \right]+l_{ox}(2R-t_{ox})\left[ 1-\exp\left( -\frac{H}{\lambda_{ox}} \right) \right]}{{(R-t)}^{2}\exp\left( -\frac{t_{ox}}{\lambda_{ox}} \right)\left[ 1-\exp\left( -\frac{H-t_{ox}}{\lambda_{Si}} \right) \right]}$, (1)

where $N$ is the number density of Si atoms, $\sigma$ is the photo-ionization cross-section, $\lambda$ is the photoelectron mean free path, subscript, *ox* and *Si*, denote the values for silicon oxide and Si^0^, respectively, and $t_{ox}$ is the thickness of the silicon oxide layer. $\frac{\sigma_{ox}}{\sigma_{Si}}$ and $\lambda_{ox}$ are assumed to be the same as those for SiO_2_ (i.e., 1.1^9^ and 3.2 nm, respectively). The number density of Si atoms in the silicon oxide layer, $N_{ox}$, is given by

$N_{ox}=\frac{\sum_{x=1}^{4} I\left( {Si}^{x+} \right)N({Si}^{x+})}{I_{ox}}$, (2)

where $I({Si}^{x+})$ and $N({Si}^{x+})$ are the area intensity of the Si 2p peak for Si^x+^ species and the number density of Si atoms for each silicon oxide species, respectively. The ratio, $\frac{N({Si}^{x+})}{N({Si}^{0})}$, is simply given by

$\frac{N({Si}^{x+})}{N({Si}^{0})}=\frac{c({Si}^{x+})}{c({Si}^{0})}\cdot\frac{7}{7+2x}$, $x=1\sim4$, (3)

where $c$ denotes the density, $c({Si}^{0})$ is taken to be 2.33 g/cm^3^ and $c$ for all silicon oxide species to be 2.20 g/cm^3^. The thickness of SiO_2_, $t_{SiO2}$, and that of suboxide species, $t_{subox}$, are defined using the following equations although the silicon oxide layer consists of mixture of SiO_2_ and suboxide species:

$t_{SiO2}=\frac{I_{SiO2}/N_{SiO2}}{I_{SiO2}/N_{SiO2}+I_{subox/N_{subox}}}$, (4)

$t_{subox}=\frac{I_{subox}/N_{subox}}{I_{SiO2}/N_{SiO2}+I_{subox/N_{subox}}}$. (5)

The thickness of the SiO_2_ layer increases monotonically with the reaction time while that of suboxide species is nearly constant at ~0.8 nm throughout the reaction. These results indicate that the following reactions proceed simultaneously:

$2Si+OH^{-}\to{Si}_{2}O+\frac{1}{2}H_{2}+e$, (6)

${Si}_{2}O+OH^{-}\to2SiO+\frac{1}{2}H_{2}+e$, (7)

$2SiO+OH^{-}\to{Si}_{2}O_{3}+\frac{1}{2}H_{2}+e$, (8)

${Si}_{2}O_{3}+OH^{-}\to2SiO_{2}+\frac{1}{2}H_{2}+e$. (9)

Generated electrons (most probably in the silicon oxide conduction band) by reactions (6)~(9) transfer to the silicon oxide surface where water molecules accept them to form OH^—^ ions and hydrogen. As explained in the text, generated hydrogen atoms from Si-based agent form hydrogen molecules in the in-vitro experiments, but it is not clear whether hydrogen molecules or hydrogen atoms play an important role in reduction of OH radicals in-vivo experiments.

**Reference**

1. Eley, D. D. & Wilkinson, P. R. Adsorption and oxide formation on aluminium films. *Proc. R. Soc. London Ser. A* **254,** 327-42 (1960).
2. Kobayashi, Y., Matsuda, S., Imamura, K. & Kobayashi, H. Hydrogen generation by reaction of Si nanopowder with neutral water. *J. Nanopart. Res.* **19**:176 (2017).
3. Himpsel FJ. McFeely FR, Taleb-Ibrahimi A. Yarmoff JA. & Hollinger G. Microscopic structure of the SiO_2_/Si interface, *Phys. Rev. B* **38,** 6084-6096 (1988).
4. Kobayashi H. Ishida T, Nakato Y. & Tsubomura H. Mechanism of carrier transport in highly efficient solar cells having indium tin oxide/Si junction, **J. Appl. Phys.** **69,** 1736-1743 (1991).
5. Shirley DA. High-resolution X-ray photoemission spectrum of the valence bands of gold, *Phys. Rev. B* **12,** 4709-4714 (1972).
6. Bozek JD. Bancroft GM. Cutler JN. & Tan KH. Vibrationally resolved core-level photoelectron spectroscopy: Si 2p levels of SiH_4_ and SiF_4_ molecules, *Phys. Rev. Lett.* **26,** 2757-2760 (1990).
7. Atkinson, A. Transport processes during the growth of oxide films at elevated temperature. *Rev. Mod. Phys.* **57,** 437-470 (1985).
8. Renault O. Marlier R. Barrett NT. Martinez E. Baron T. Gely M. & De Salvo B. Modeling the XPS Si 2p core-level intensities of silicon nanocrystals for determination of oxide shell thickness, *Surf. Interface Anal*. **38,** 486-488 (2006). doi: 10. 1002/sia.2223
9. Hochella, Jr. MF. & Carim AH. A reassessment of electron escape depths in silicon and thermally grown silicon dioxide thin films, *Surf. Sci.* **197,** L260-268 (1988).

Supplementary Fig. 1 **Schematic model of Si-based agent.**  OH^－^ ions in the solution are adsorbed on the surface of silicon oxide, generating electrical field in the silicon oxide layer. Inward migration of OH^－^ ions is enhanced by the electrical field (step 1). When OH^－^ ions reach the silicon oxide/Si interface, interfacial reaction proceeds (step 2). Generated electrons at the interface transfer to the surface of silicon oxide (step 3), and then water molecules accept them (step 4).

Supplementary Fig. 2 **XPS spectra in the Si 2p region for Si-based agent**. Spectrum (**a**) is measured before the reaction, and spectra (b), (c), and (d) were recorded after the hydrogen generation reaction under pH 8.3 and 36ºC for the following periods: (**b**) 1 h, (**c**) 6 h, (**d**) 24 h. X-ray photoelectron spectroscopy (XPS) spectra were measured by use of a KRATOS AXIS-165x spectrometer with an Mg Kα radiation source. Photoelectrons were collected in the surface-normal direction.

Supplementary Fig. 3 **Thickness of the silicon oxide layer on Si-based agent.** The thickness of the SiO_2_ layer (**a**) and that of the silicon suboxide layer (**b**) vs. the reaction time are obtained by deconvolution of the XPS spectra of Supplementary Fig. 2.


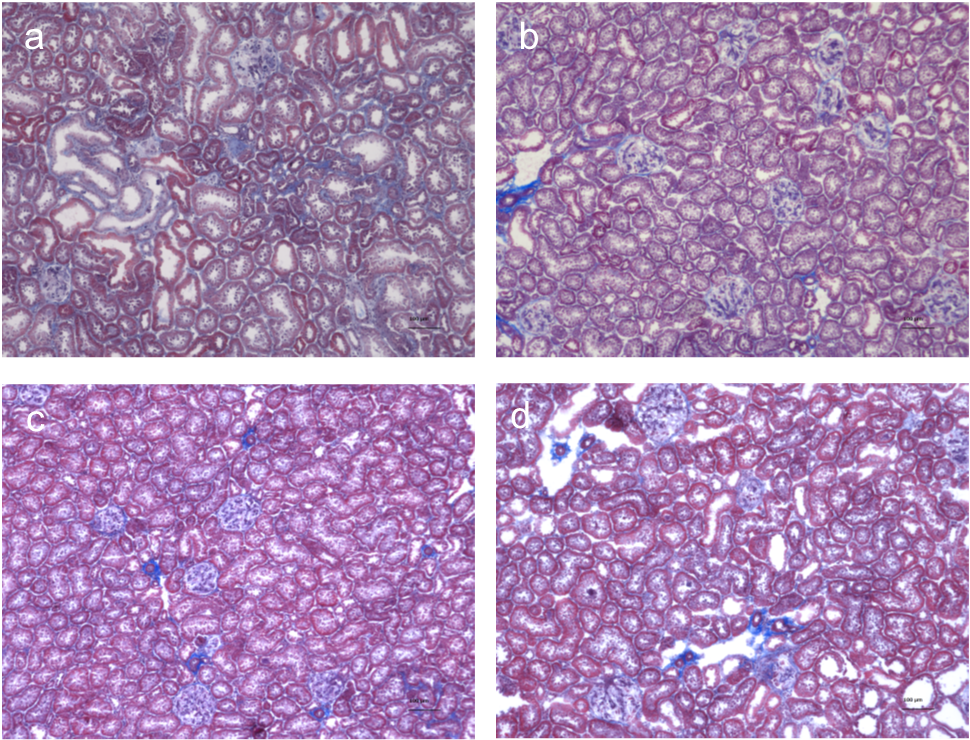


100μm

100μm

100μm

100μm


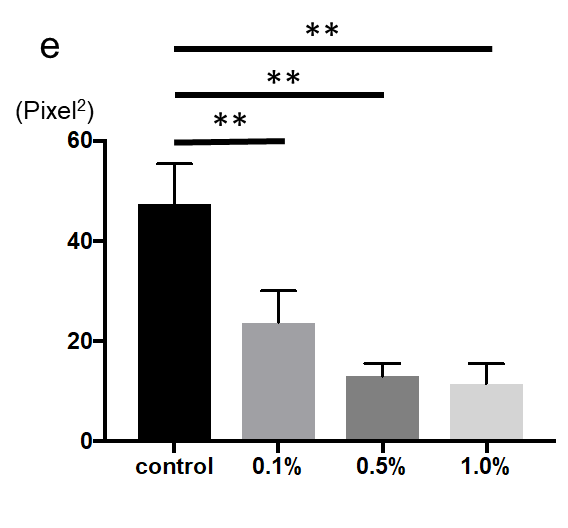


Supplementary Fig. 4  **Effect on interstitial fibrosis.** The interstitial fibrotic area was stained blue on Masson's trichrome in the control group (a) or the Si-based agent (0.1wt.%: b, 0.5wt.%: c, 1.0wt.%: d) groups, respectively. Scale bar: 100µm. The interstitial fibrotic area was quantitatively assessed using a color image analyzer (e). (**p < 0.01)


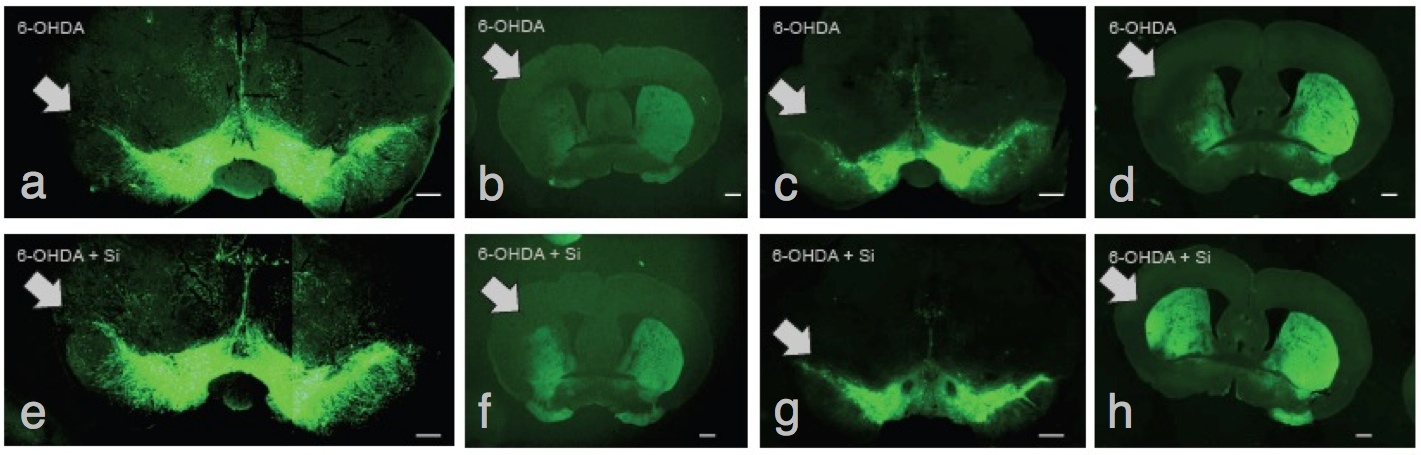


Supplementary Fig. 5

Supplementary Fig. 5. Additional tyrosine hydroxylase staining in the mouse brain showing effects of Si-based agent on the 6-OHDA-induced dopaminergic toxicity. The micrographs of tyrosine hydroxylase immunoreactivities for substantia nigra pars compacta (a, c, e, g) and Striatum (b, d, f, h). White arrow indicated lesion site. Upper images: 6-OHDA group (a-d); lower images: 6-OHDA + Si-based agent group (e-h). Scale bar: 200µm (a, c, e, g) and 1mm (b, d, f, h).
